# Fullerene-Supported Single-Atom Catalysts for Electrocatalytic Water Splitting: Progress, Challenges, and Machine Learning Perspectives

**DOI:** 10.3390/molecules30234494

**Published:** 2025-11-21

**Authors:** Chun-Xiang Li, Shu-Ling Tong, De-Sheng Ma, Hao Huang, Xiao-Nan Zheng, Yu-Ping Zhang, Hong-Yan Jiao, Ling-Bo Qu, Cheng-Xing Cui

**Affiliations:** 1 College of Food Science and Technology, Henan University of Technology, Zhengzhou 450001, China; chunxiang.li@mail.bnu.edu.cn; 2 Postdoctoral Research Base, School of Chemistry and Chemical Engineering, Institute of Computational Chemistry, Henan Institute of Science and Technology, Xinxiang 453003, China; 3 School of Chemistry and Materials Engineering, Hunan University of Arts and Science, Changde 415000, China; 4 School of Information Engineering, Xinxiang Institute of Engineering, Xinxiang 453700, China

**Keywords:** fullerene SACs, water splitting, HER, OER, machine learning

## Abstract

Fullerene-supported single-atom catalysts (SACs) have emerged as a promising class of materials for electrocatalytic overall water splitting, offering a route to reduce reliance on scarce and costly precious metals. This review systematically summarizes recent advances in the design, synthesis, and application of fullerene-based SACs, with an emphasis on their unique structural, electronic, and catalytic properties. The exceptional stability, conductivity, and surface chemistry of fullerenes enable strong interactions with metal atoms, allowing high dispersion and enhanced catalytic performance for both the hydrogen evolution reaction (HER) and oxygen evolution reaction (OER). Recent studies demonstrate that C_60_- and C_24_-based materials, when combined with transition metals such as Pt, Ru, and V, exhibit superior HER/OER activity, bifunctionality, and spin-selective catalytic pathways. The vast structural space of fullerene–metal combinations presents new opportunities, which can be efficiently explored using machine learning and high-throughput simulations. By integrating density functional theory, transition state modeling, and data-driven techniques, this emerging research frontier is paving the way for rational catalyst design. The review concludes by proposing a machine learning-assisted framework to predict and screen high-performance fullerene-based SACs, ultimately accelerating the development of efficient, stable, and scalable electrocatalysts for sustainable hydrogen production.

## 1. Introduction

With the increasing energy demand, hydrogen energy is regarded as an ideal sustainable energy option due to its high calorific value, zero carbon emissions, and excellent storage and transportation properties [1]. Water electrolysis has attracted significant attention due to its environmentally friendly advantages. However, current commercial electrolysis catalysts are still dominated by precious metals, particularly platinum-group metals (e.g., platinum (Pt), iridium (Ir), and ruthenium (Ru)) [2,3,4,5,6]. Among them, Pt/C catalysts account for up to 50% of the HER market share for overall water splitting, while IrO_2_ and RuO_2_ dominate over 80% of the OER market [5,7,8]. Precious metal catalysts are prone to oxidation, dissolution, or agglomeration under strongly acidic/alkaline conditions or high potentials, leading to degraded catalytic activity [1]. For instance, Pt tends to deactivate in acidic HER environments due to excessive hydrogen adsorption, requiring frequent regeneration. Hydrogen production through water electrolysis has become a cornerstone of the sustainable hydrogen economy, providing a clean and efficient route to convert renewable electricity into chemical energy [1,2]. Recent progress in electrocatalyst design for the hydrogen and oxygen evolution reactions (HER and OER) has significantly enhanced efficiency and durability. In particular, single-atom catalysts (SACs) have attracted wide attention owing to their maximal atomic utilization and precisely tunable active sites [3,4,5].

Moreover, the recycling processes for precious metals are complex and costly, often involving highly corrosive reagents (e.g., aqua regia), which further escalate both economic and environmental burdens [1,4,7]. Consequently, despite their exceptional catalytic activity and stability, the widespread application of precious metal catalysts is severely constrained by their exorbitant costs and resource scarcity. Particularly concerning are platinum-group metals (PGMs), with global reserves estimated at merely 70,000 tons. China’s share constitutes less than 1% of these reserves, resulting in an alarming 80% import dependency. This heavy reliance leads to volatile catalyst prices, significantly impeding the stable development of China’s water electrolysis industry [9]. Globally, substantial progress has been made in both acidic and alkaline electrolyzers toward developing cost-effective and robust catalysts to replace noble metals [6,7,8]. Current frontiers emphasize single-atom catalysis, heterostructure engineering, and carbon-based supports such as graphene, MXenes, and fullerenes to optimize activity and long-term stability. The integration of theoretical simulations with machine learning has further accelerated catalyst screening and mechanistic understanding, enabling rapid discovery of highly active HER and OER materials [6,8].

Carbon-based materials, including graphene, carbon nanotubes, and graphitic carbon nitride, have been extensively used as SAC supports because of their high conductivity and tunable surface chemistry [10,11,12]. However, these systems often suffer from metal aggregation and nonuniform active sites. In contrast, fullerene (C_60_) and its derivatives provide a unique zero-dimensional framework with uniform geometry and strong electron-accepting capability, offering distinct advantages for stabilizing isolated metal atoms and modulating their electronic structure [13,14,15].

Enhancing the atomic utilization efficiency of precious metals, developing non-precious metal catalysts, and achieving breakthroughs in atomic-level active site optimization are crucial for reducing heavy reliance on precious metals and promoting the sustainable development of water electrolysis hydrogen production. To address these challenges, researchers have recently explored the potential of zero-dimensional carbon materials, especially fullerenes, as promising supports for single-atom catalysts in electrocatalytic water splitting. Notably, several experimental studies have already demonstrated that fullerene-supported single-metal atoms can effectively catalyze hydrogen and oxygen evolution reactions, highlighting their great potential as alternatives to precious-metal-based systems [10,13,15]. In this review, we systematically discuss and analyze both experimental advances and theoretical insights into fullerene-supported single-atom catalysts for water splitting, and further propose the integration of machine learning with theoretical calculations as a powerful strategy to accelerate the discovery and design of next-generation fullerene-based electrocatalysts.

According to Kment et al., comprehensive reviews have already been conducted on single-atom catalysts based on earth-abundant metals for energy conversion, including electrochemical and photocatalytic water splitting [16]. In addition, Cao et al. reported a significant experimental advance in constructing high-density SACs for overall water splitting at industrial current densities, highlighting the maturity and rapid progress of this research field [17]. In contrast, systematic reviews focusing specifically on fullerene-supported single-atom catalysts for water splitting remain very scarce, with only a few scattered studies addressing their structures and catalytic mechanisms without a comprehensive summary of their overall progress and challenges. Fullerenes, with their well-defined molecular geometry, tunable electronic properties, and strong ability to stabilize isolated metal atoms, offer distinctive advantages compared to other carbon supports. Therefore, this review provides a comprehensive overview of recent progress in fullerene-supported SACs, including experimental and theoretical studies, to elucidate their structural characteristics, synthesis strategies, and catalytic mechanisms. Moreover, perspectives on future design concepts—such as functionalization, polymerized fullerenes, and machine learning-assisted discovery—are presented to guide further development in this emerging research area [18,19].

## 2. Application Potential of Fullerenes in Water Splitting Catalysis

### 2.1. Zero-Dimensional Carbon Materials for HER and OER

Fullerenes, as zero-dimensional carbon materials with well-defined molecular structures, have recently been successfully applied in catalytic water splitting reactions through the synthesis of monolayer fullerene networks using appropriate interlayer bond-breaking strategies. The introduction of metal species can further enhance the catalytic efficiency of H_2_ production from water [10,11,12,13,14,15].

**Figure 1:**
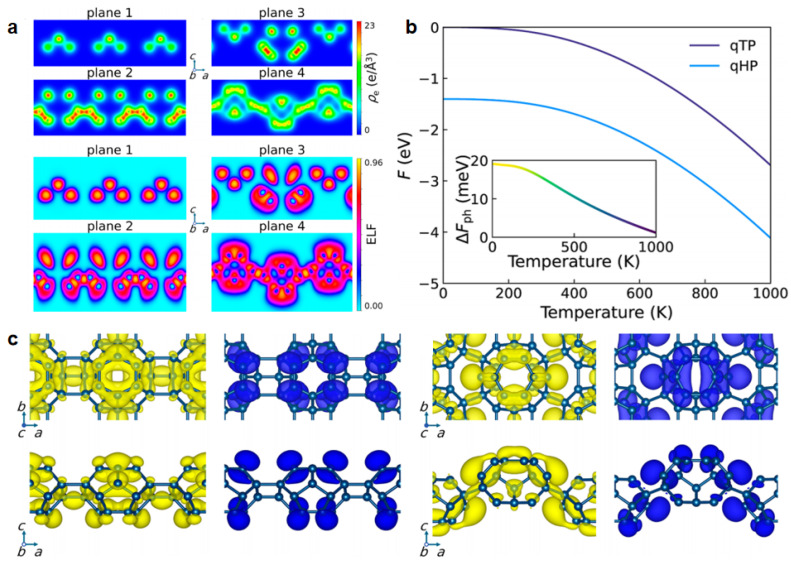
(**a**) Charge density on the (010) plane illustrating intermolecular bonding features, together with their corresponding electron localization functions. (**b**) Free energy curves of qTP and qHP C_24_ monolayers per unit, with the free energy of qTP at 0 K set as reference, and the phonon free energy difference (ΔFph) shown in the inset. (**c**) Top and side views of VBM (yellow) and CBM (navy) charge densities contributing most to the first bright exciton for qTP and qHP C_24_, with the VBM of qTP C_24_ being a superposition of doubly degenerate states. Reproduced from Ref. [13] with permission from American Chemical Society.

As shown in Figure 1a,b, Wu and Peng [13] present a theoretical study on two-dimensional (2D) networks constructed from the smallest stable [5,6] fullerene cage, C_24_, exploring their structural stability and photocatalytic potential for water splitting. Unlike traditional carbon materials composed of atomic lattices, these fullerene-based superatomic lattices leverage the unique bonding characteristics of C_24_ molecules to form highly cohesive, thermodynamically favorable monolayer structures. Two types of monolayer C_24_ networks were proposed: a quasi-tetragonal phase (qTP) and a quasi-hexagonal phase (qHP). Both show higher cohesive energies and mechanical strength compared to the more commonly studied polymeric C_60_ monolayers. First-principles density functional theory (DFT) calculations reveal that the qHP structure is energetically preferred at all temperatures due to its denser packing and stronger inter-fullerene bonding. Phonon dispersion analyses and elastic constant evaluations confirm the dynamic and mechanical stability of both configurations, with qHP exhibiting greater resistance to shear and deformation. Electronic structure calculations using hybrid functionals indicate that monolayer C_24_ possesses large band gaps (3.1–3.7 eV), comparable to TiO_2_, a benchmark photocatalyst. These band gaps straddle the redox potentials of water in both acidic and neutral pH conditions, making them suitable candidates for photocatalytic overall water splitting. Time-dependent DFT simulations show strong optical absorption in the visible to UV-A range, driven by bright excitonic transitions with high binding energies. Additionally, monolayer C_24_ materials support type-II band alignment in heterostructures, enabling effective spatial separation of photoexcited electrons and holes. Critically, the C_24_ networks offer a significantly higher density of thermodynamically active sites for hydrogen evolution-up to three times more than C_60_ analogs—especially under mildly acidic conditions (pH > 3). As shown Figure 1c, free energy calculations of hydrogen adsorption across symmetry-irreducible sites confirm the viability of both Volmer–Tafel and Volmer–Heyrovsky reaction pathways, with photoexcitation facilitating spontaneous H_2_ formation across nearly all sites. This work establishes C_24_-based monolayers as promising, tunable platforms for photocatalytic applications and provides a path toward engineering carbon-based 2D materials with targeted optoelectronic and catalytic functionalities. By leveraging the modularity of fullerene building blocks, the study opens new opportunities in solar energy conversion and hydrogen fuel generation.

Wang et al. [15] report the synthesis and photocatalytic application of a few-layer C_60_ network capable of splitting pure water into hydrogen (H_2_) and hydrogen peroxide (H_2_O_2_) under visible light irradiation, without the use of sacrificial reagents. This represents a significant advance in carbon-based photocatalysts, combining clean hydrogen production with the generation of valuable H_2_O_2_ through a kinetically favorable two-electron water-splitting pathway. The few-layer fullerene networks were fabricated via exfoliation of Mg_4_C_60_ bulk crystals using an organic cation slicing technique. Characterization through SEM, TEM, AFM, XRD, Raman spectroscopy, and NMR confirmed the successful formation of few-layer C_60_ nanosheets with high crystallinity, enhanced surface area, and well-preserved structural integrity. Optical and electrochemical analyses revealed that these 2D C_60_ nanosheets have a bandgap (~2.05 eV) and suitable band edge positions that thermodynamically support the overall water-splitting reaction. Mott–Schottky plots and band structure diagrams further confirmed their ability to drive both H^+^ reduction to H_2_ and the oxidation pathway yielding H_2_O_2_. Photocatalytic experiments showed that under visible-light illumination (λ > 420 nm), the few-layer C_60_ network achieved H_2_ and H_2_O_2_ production rates of 91 and 116 μmol g^−1^ h^−1^, respectively. Control experiments using bulk Mg_4_C_60_ or pristine C_60_ exhibited negligible activity, underscoring the importance of exfoliation and nanoscale morphology. EPR and mass spectrometry measurements confirmed that reactive oxygen species such as *OH and *O_2_^−^ intermediates played key roles in the H_2_O_2_ generation mechanism. Additional experiments under varying atmospheric conditions (N_2_, air, O_2_) revealed that O_2_ reduction by photogenerated electrons contributes to enhanced H_2_O_2_ yield in oxygen-rich environments.

**Figure 2:**
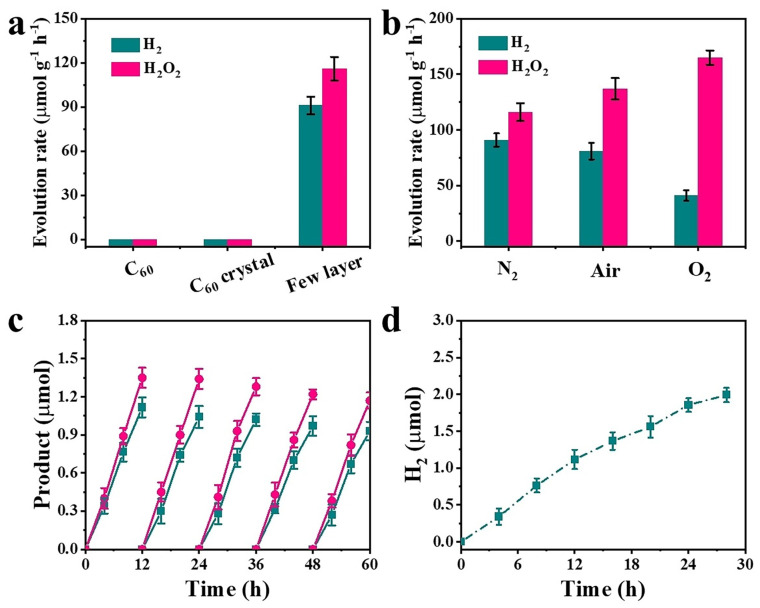
(**a**) Comparison of H_2_ and H_2_O_2_ evolution rates for C_60_, Mg_4_C_60_, and few-layer samples. (**b**) H_2_ and H_2_O_2_ evolution of few-layer C_60_ under different atmospheres. (**c**) Cyclic stability of H_2_ and H_2_O_2_ evolution. (**d**) Long-term H_2_ stability of few-layer C_60_ in N_2_ atmosphere. Reproduced from Ref. [15] with permission from Wiley.

As shown Figure 2, charge separation and transfer efficiency were significantly improved in the few-layer networks compared to bulk Mg_4_C_60_, as demonstrated by electrochemical impedance spectroscopy (EIS) and transient photocurrent response. Long-term and cyclic stability tests showed that the few-layer C_60_ samples maintained substantial photocatalytic performance for over 24 h, with some decline due to nanosheet agglomeration, which was visually confirmed post-reaction. This work demonstrates the dual capability of few-layer C_60_ nanostructures to produce both H_2_ and H_2_O_2_ from pure water via a sustainable, metal-free photocatalytic route. The fullerene-based architecture offers a promising avenue for designing efficient, scalable, and multifunctional 2D carbon photocatalysts for integrated solar fuel and chemical production systems.

### 2.2. Enhancement via Metal Introduction

Fullerenes possess unique spherical structures with *sp^2^*-hybridized carbon surfaces, excellent conductivity, and remarkable chemical stability. These characteristics enable strong covalent bonding with metal atoms (e.g., Pt and V) through π-type coordination, effectively preventing metal aggregation while facilitating electron transfer. This interaction forms fullerene-supported SACs with superior catalytic properties [18,19].

**Figure 3:**
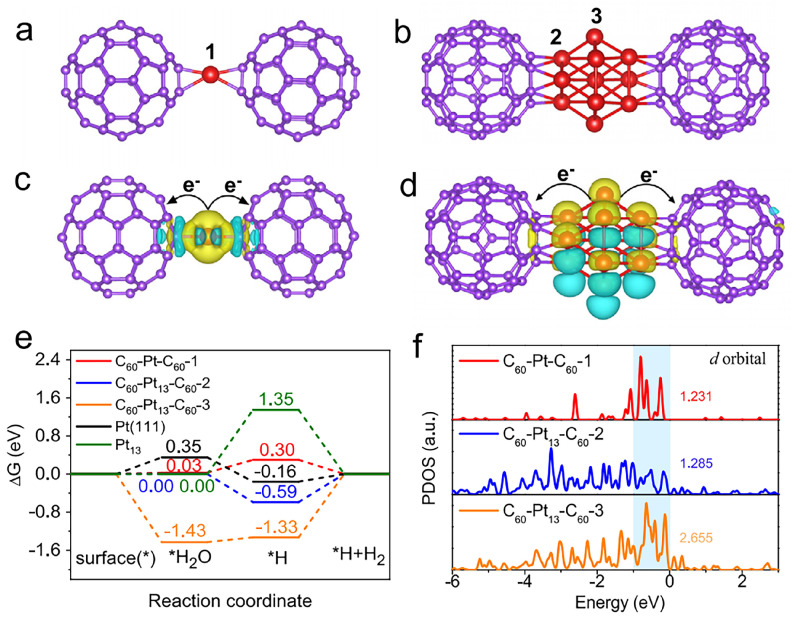
(**a**) Side view of optimized model for a Pt single atom. (**b**) Side view of small Pt clusters. (**c**) Charge density map of C_60_-Pt-C_60_. (**d**) Charge density map of C_60_-Pt_13_-C_60_ (blue = electron loss, yellow = electron gain). (**e**) Adsorption energies of H_2_O and H on different Pt-C_60_ systems, Pt (111), and Pt_13_ cluster. (**f**) PDOS of Pt atoms in C_60_-Pt-C_60_-1, C_60_-Pt_13_-C60-2, and C_60_-Pt_13_-C_60_-3. *H_2_O and *H represent the adsorbed states of H_2_O and H. respectively. Reproduced from Ref. [18] with permission from Springer Nature.

Zhang et al. [18] report the synthesis and characterization of a high-loading single-atomic platinum catalyst supported on fullerene C_60_ (Pt/C_60_) for efficient HER in alkaline media. Recognizing the limitations of traditional Pt-based catalysts—including low single-atom dispersion, high synthesis temperatures, and insufficient utilization efficiency—the authors designed a novel Pt–C_60_ polymeric structure formed through a mild room-temperature reaction. In this system, platinum atoms are anchored between two curved C_60_ molecules via η^2^-π bonding, enabling a high Pt loading of over 21 wt% while maintaining atomic dispersion. Structural characterizations using XPS, XAS, and HAADF-STEM confirmed the predominance of Pt single atoms with minimal aggregation. Electrochemical measurements revealed that Pt/C_60_-2 exhibits exceptional HER activity with an ultra-low overpotential of 25 mV at 10 mA cm^−2^ in 1 M KOH—significantly outperforming commercial 20 wt% Pt/C—and maintains high stability over 100 h of operation. Further investigations demonstrated that the catalyst operates via a Volmer–Heyrovsky mechanism with enhanced kinetics, as evidenced by a low Tafel slope and high turnover frequency (TOF). As shown in Figure 3, DFT calculations revealed that the curved C_60_ surface induces a shell-like charge redistribution around the Pt atoms, generating a built-in electric field that promotes water adsorption and facilitates hydrogen desorption. The optimized *H and *H_2_O adsorption energies at the Pt-C_60_ interface are consistent with the Sabatier principle and contribute to the outstanding catalytic performance. This study underscores the potential of zero-dimensional carbon supports such as C_60_ in stabilizing and electronically tuning single metal atoms for electrocatalysis. The combination of high atomic utilization, strong metal–support interactions, and favorable electronic structure engineering offers a new strategy for developing cost-effective and durable catalysts for sustainable hydrogen production.

## 3. Progress of Fullerene-Supported SACs

### 3.1. Fullerene-Pt and Fullerene-V Systems

**Figure 4:**
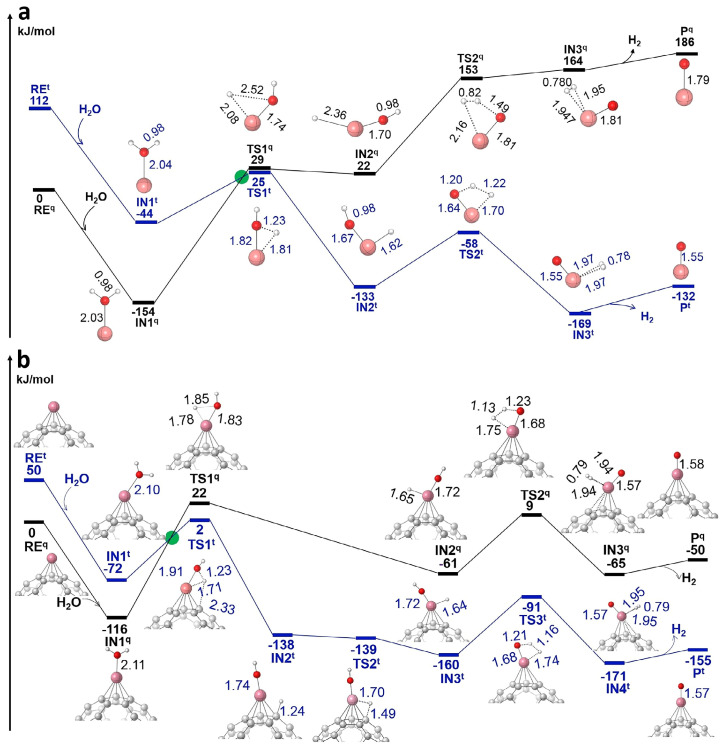
Calculated reaction potential energy surfaces (PESs) for (**a**) V^+^ + H_2_O→V^+^ (H_2_O)→VO^+^ + H_2_ and (**b**) C_60_V^+^ + H_2_O→C_60_V^+^ (H_2_O)→C_60_VO^+^ + H_2_ with η^5^ C_60_V^+^ as starting point at BPW91/6–311++G(2df,2p)//6–31G(d,p) level of theory. Both quintet (black) and triplet (blue) surfaces are shown. The various states are denominated with RE for reactant, IN for reaction intermediate, TS for transition state, and P for product, and labeled with a superscript t and q, for triplet or quintet, respectively. The quintet-to-triplet spin-crossing points, i.e., the Minimum Energy Crossing Points (MECPs), are schematically indicated with green circles at the crossings of the two potential energy surfaces. Key bond lengths are indicated in Å. All energies are given with respect to those of the reactants on the quintet surface. Reproduced from Ref. [19] with permission from Wiley.

Recently, research by Professor Gaolei Hou’s group at Xi’an Jiaotong University demonstrated that the surface sites and metal-loading configurations of V^+^-supported C_60_ catalysts significantly influence the water-splitting reaction mechanism. Their work revealed that metal coordination can substantially reduce reaction energy barriers, thereby enhancing catalytic efficiency [19,20]. Hou et al. [19] investigated the catalytic role of a vanadium single atom supported on a C_60_ fullerene (C_60_V^+^) in driving water splitting reactions. As shown Figure 4a,b, using infrared multiple photon dissociation (IRMPD) spectroscopy and density functional theory (DFT) calculations, they characterized the intermediates and mapped the reaction pathway of the process C_60_V^+^ + H_2_O→C_60_VO^+^ + H_2_. The study demonstrates that the C_60_ support significantly lowers the reaction barrier—by more than 70 kJ/mol—compared to an isolated V^+^ ion. This effect is attributed to both orbital overlap between the C_60_ surface and a hydrogen atom from water and to charge transfer from the support to the metal center, which stabilizes the reaction transition states. Spin state transitions (quintet to triplet) play a key role in enabling the otherwise spin-forbidden reaction steps. The C_60_ support also temporarily hosts hydrogen atoms during the reaction, acting as a “hydrogen shuttle”, which facilitates H_2_ formation and release. This work provides molecular-level insights into the essential role of carbon-based supports in single-atom catalysis and highlights how support-induced effects can be leveraged to improve the efficiency of water-splitting catalysts for clean hydrogen production.

### 3.2. Bifunctionality and Spin-State Modulation

**Figure 5:**
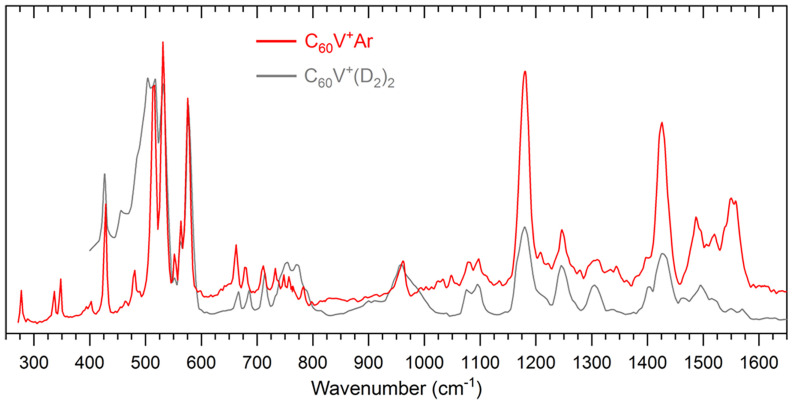
IRMPD spectra of Ar-tagged C_60_V^+^ and D2-tagged C_60_V^+^ for comparison. Reproduced from Ref. [20] with permission from American Chemical Society.

Previous studies have predominantly focused on either the HER or OER half-reactions separately, revealing that HER catalysts typically perform better in acidic conditions while OER catalysts favor alkaline environments. However, C_60_-based SACs demonstrate exceptional bifunctional catalytic capabilities, simultaneously facilitating both OER and HER. This unique property enables them to drive the complete water-splitting process involving the challenging four-electron transfer mechanism [20,21]. Xu et al. [20] investigate the binding site of a single vanadium cation (V^+^) on the surface of buckminsterfullerene (C_60_) using a combination of advanced spectroscopic and theoretical techniques. As shown Figure 5, the C_60_V^+^ complex was synthesized in the gas phase and characterized via infrared multiple photon dissociation (IRMPD) spectroscopy, assisted by messenger-tagging with Ar to reduce spectral perturbations. High-resolution experimental spectra were compared to theoretical IR spectra derived from DFT calculations, with various possible binding sites (η^5^, η^6^, η^2(6−6)^, η^2(5−6)^) and spin states considered. Among them, the η^2^ configuration—where the V^+^ sits above a pentagonal face of C_60_—was identified as the most probable structure, supported by both spectral cosine similarity analysis and calculated relative energies. Born–Oppenheimer molecular dynamics (BOMD) simulations at elevated temperatures further confirmed the thermal stability of the η^5^ isomer and its dominance under experimental conditions. Energy decomposition analysis (EDA-NOCV) revealed that the strong binding of V^+^ arises mainly from significant orbital interactions and electrostatic contributions, with η^5^ C_60_V^+^ exhibiting the largest bond dissociation energy among the isomers. Detailed orbital interaction analysis showed a mixture of dative bonding from C_60_ to V^+^ and back-donation from V^+^ to the fullerene, indicating rich electronic interactions. This comprehensive experimental-theoretical approach not only clarifies the precise adsorption site of a transition metal on C_60_ but also highlights the pivotal role of orbital interactions in stabilizing metal–fullerene complexes, providing critical insights for designing single-atom catalysts and functional fullerene-based materials.

**Figure 6:**
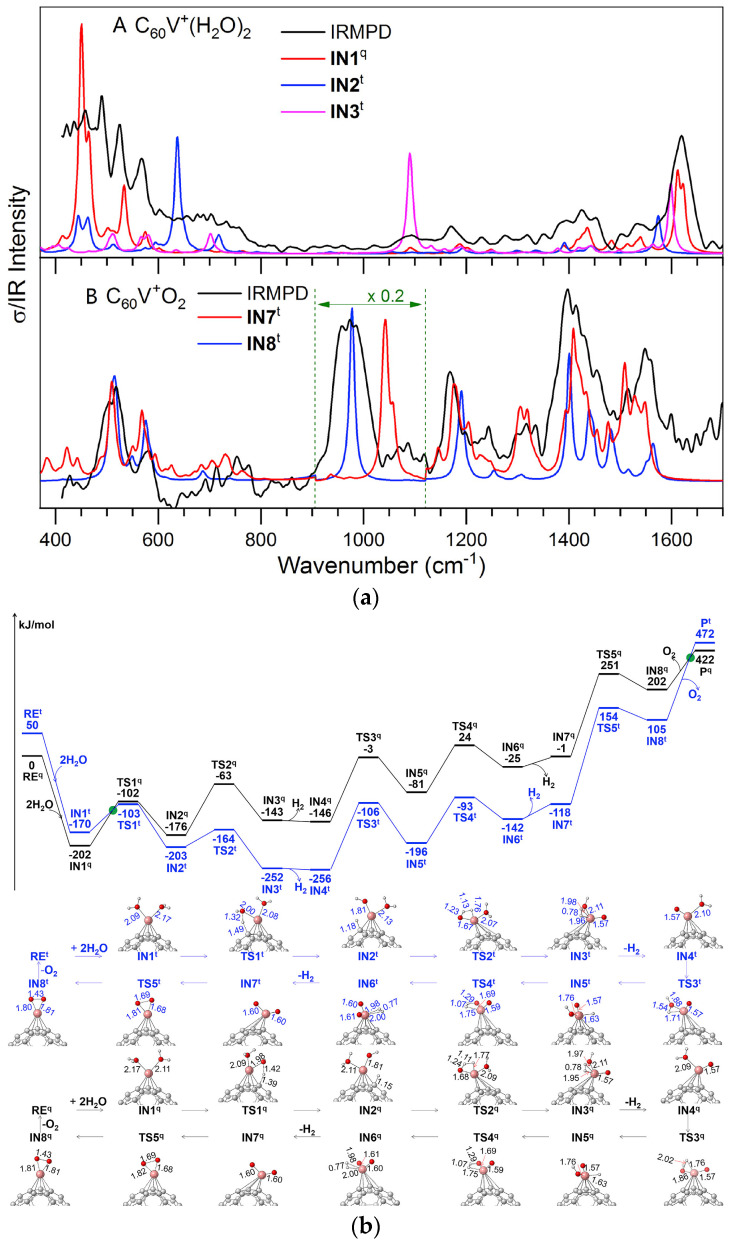
(**a**) IRMPD spectra of the complexes with nominal chemical formulas of C_60_V^+^(H_2_O)_2_ and C_60_V^+^O_2_, and the calculated spectra of η^5^ IN1^q^, IN2^t^, IN3^t^, IN7^t^, and IN8^t^ at the BPW91/6-31G(d,p) level of theory. (**b**) Calculated reaction pathways for 2H_2_O→2H_2_ + O_2_ catalyzed by η^5^C_60_V^+^ in both quintet (black) and triplet (blue) states at BPW91/6-311++G(2df,2p)//6-31G(d,p) level of theory. Reproduced from Ref. [21] with permission from Elsevier.

Li et al. [21] investigated the catalytic mechanism of water splitting mediated by C_60_-supported vanadium single atoms (C_60_V^+^), combining theoretical calculations and IRMPD spectroscopy to identify key intermediates and electronic behavior. The study reveals that while isolated V^+^ ions cannot complete the catalytic cycle due to geometric constraints, the presence of the C_60_ support enables full water splitting, producing both H_2_ and O_2_. The C_60_ cage plays a dual role: geometrically, it acts as a “hydrogen shuttle” facilitating hydrogen atom transfer; electronically, it behaves as an “electron sponge” that stabilizes multiple oxidation states of the vanadium center throughout the reaction cycle. Key intermediates such as C_60_V^+^(H_2_O)_2_ and C_60_V^+^O_2_ were confirmed through IRMPD spectroscopy, with theoretical calculations supporting a multi-step reaction mechanism. As shown in Figure 6a,b, spin-state transitions and orbital interactions between V-d orbitals and the C_60_ framework were found to significantly lower energy barriers for key steps in the catalytic process. This work emphasizes the importance of support materials not only as structural anchors but also as active electronic participants, and provides fundamental insight into designing efficient single-atom catalysts for hydrogen production via overall water splitting.

Further studies have revealed that C_60_ can form diverse coordination structures with various metal atoms or clusters, enabling precise tuning of metal–fullerene interactions to synergistically enhance both HER and OER performance. Representative examples include: (i) Ru-based C_60_(OH)_24_ catalysts demonstrating exceptional alkaline HER activity, and (ii) Pt/C_60_ systems that facilitate water adsorption and hydrogen desorption, thereby accelerating HER kinetics [22,23]. Lee et al. [22] systematically investigated the coordination chemistry between Buckminsterfullerene (C_60_) and various transition metal clusters, revealing a series of novel bonding modes and rich electronic interactions that have significant implications for materials design and molecular electronics. Their study focuses on the formation of exohedral metallofullerene complexes, particularly those featuring C_60_ as a π- or σ-donating ligand to metal cluster cores such as Os_3_, Re_3_, Ru_3_, and Rh_6_. The research demonstrates that C_60_ can engage in multiple bonding configurations, including η^2^-C_60_, μ-η^2^:η^2^-C_60_, and μ_3_-η^2^:η^2^:η^2^-C_60_ modes. Among these, the μ_3_-η^2^:η^2^:η^2^-C_60_ “face-capping” interaction, where three metal atoms simultaneously bind to three adjacent double bonds of a single C_60_ ring, was found to be particularly significant. These complexes exhibit high structural stability and allow for extensive ligand substitution on the metal core without compromising the fullerene interaction. Ligand-induced transformation between bonding modes, such as from π-type to σ-type coordination (e.g., μ_3_-η^1^:η_2_:η^1^ or μ_3_-η^1^:η^1^:η^2^), was also observed, showing C_60_’s exceptional versatility as a ligand. Importantly, electrochemical studies revealed unusually strong electronic communication between the C_60_ moiety and the metal cluster framework. For example, in μ_3_-η^2^:η^2^:η^2^-C_60_ complexes, electron transfer between C_60_ and the metal core was found to be highly efficient, as evidenced by shifted reduction potentials and enhanced redox stability. Furthermore, the incorporation of multiple C_60_ units onto a single cluster framework (such as the Rh_6_ sandwich complex with two face-capping C_60_ ligands) demonstrated strong inter-fullerene communication mediated by the metal core. These findings suggest that the electronic properties of both the C_60_ and the metal center can be finely tuned by careful ligand and framework design. This study provides deep insights into the structural dynamics and redox behavior of C_60_–metal cluster complexes. It establishes C_60_ not merely as a passive support, but as an electronically active and structurally adaptive ligand that can modulate metal-centered properties. These discoveries open up avenues for designing C_60_-based molecular materials with tailored electronic communication and potential utility in molecular electronics, nanoscale catalysis, and carbon-based device integration.

**Figure 7:**
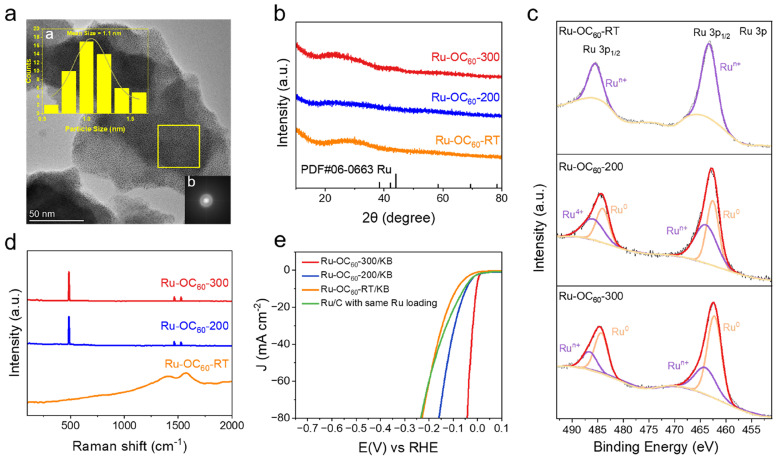
(**a**) TEM image of Ru-OC_60_-RT with particle size distribution and FFT of the selected area, where a shows the particle size distribution of Ru particles and the corresponding FFT pattern of the region marked by the yellow square, while b shows the corresponding FFT pattern of the selected region, indicating amorphous diffraction rings. (**b**) XRD patterns of Ru-OC_60_ and Ru/C samples. (**c**) High-resolution Ru 1s XPS spectra. (**d**) Raman spectra of Ru-OC_60_ and Ru/C. (**e**) HER polarization curves comparing Ru-OC_60_ at different treatments with Ru/C. Reproduced from Ref. [23] with permission from American Chemical Society.

**Figure 8:**
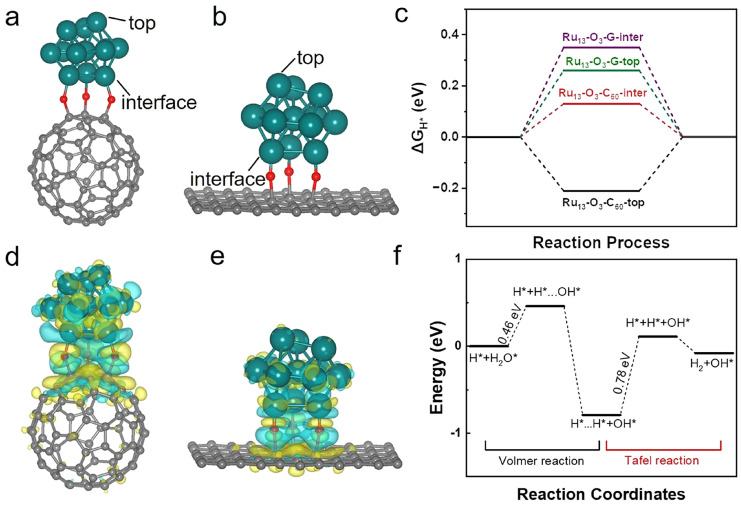
(**a**) Side view of Ru_13_-O_3_-C_60_. (**b**) Side view of Ru_13_-O_3_-graphene. (**c**) Calculated ΔG_H*_ values at different active sites of Ru_13_-O_3_-C_60_ and Ru_13_-O_3_-G. (**d**) Charge density map of Ru_13_-O_3_-C_60_. (**e**) Charge density map of Ru_13_-O_3_-G (cyan = electron loss, yellow = electron gain). (**f**) Volmer–Tafel energy diagram of Ru_13_-O_3_-C_60_, where * indicates an adsorbed state. Reproduced from Ref. [23] with permission from ACS.

Li et al. developed a high-performance HER catalyst by anchoring Ru nanoparticles onto C_60_ fullerenol, forming a Ru-O-C_60_ interface that enhances both stability and activity in alkaline media [23]. The C_60_ fullerenol serves as a support and electronic modulator, enabling ultra-high Ru loading (38.6 wt%) while maintaining uniform nanoparticle dispersion and preventing aggregation. The electron-withdrawing nature of C_60_ induces electron transfer from Ru to the fullerene, which weakens Ru-H bonding and promotes water dissociation, key steps in the HER process. The resulting Ru-OC_60_ catalyst exhibited an ultra-low overpotential (4.6 mV at 10 mA cm^−2^) and a low Tafel slope (24.7 mV dec^−1^), outperforming commercial Pt/C and other reported Ru-based systems. DFT calculations confirmed that the engineered Ru-O-C_60_ interface optimizes hydrogen adsorption and accelerates the Volmer–Tafel HER pathway. As shown in Figure 7, moreover, the catalyst demonstrates excellent durability, nearly 100% Faradaic efficiency, and superior performance across different pH conditions. As shown in Figure 8, this work highlights the potential of C_60_ fullerenol as a versatile support for noble-metal nanoparticles and offers a promising route for designing stable, cost-effective electrocatalysts for sustainable hydrogen production.

**Figure 9:**
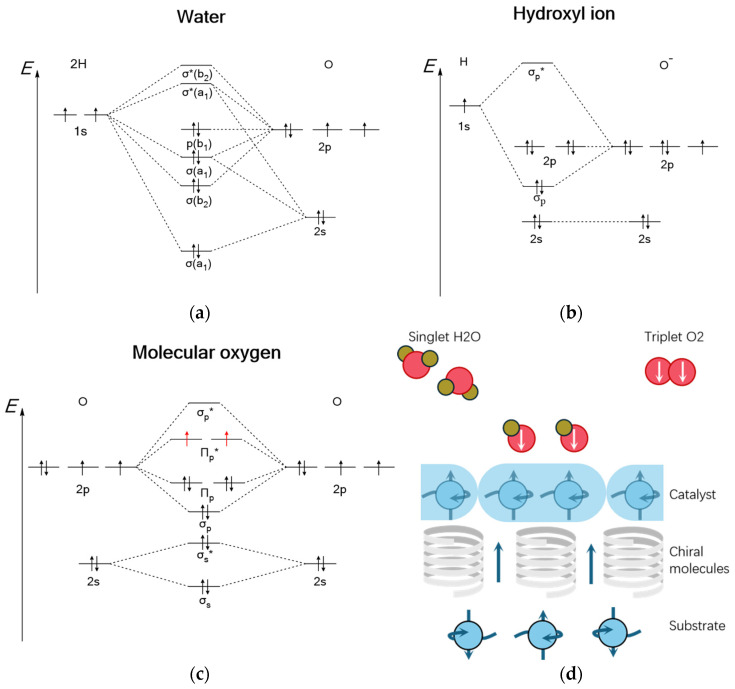
(**a**) Spin-state evolution during OER. σ* indicates antibonding molecular orbital. (**b**) Chiral spin catalyst mechanism with spin–orbit coupling. (**c**) Electron spin polarization from CISS effect. (**d**) Strategy of spin regulation to accelerate OER kinetics in fullerene-based SACs.

Moreover, as illustrated in Figure 9, the spin-state barrier during water-to-oxygen conversion in OER can be effectively addressed through Chiral-induced spin selectivity (CISS), which regulates electron spin states to optimize OER kinetics [24]. Recently, the R. Nitschke group at the University of Cambridge developed a cavity-confined strategy to direct selective Diels–Alder reactions between C_60_ and cage edges, achieving precise synthesis of chiral fullerene adducts containing six stereocenters. This breakthrough provides a fundamental platform for implementing CISS in fullerene-based SACs [25]. Consequently, the rational design of fullerene-supported SACs, leveraging both efficient electron exchange between fullerenes and metal centers and CISS-mediated spin-state control, represents a promising strategy to simultaneously optimize the thermodynamic and kinetic processes of both HER and OER. This approach may ultimately overcome the current reliance on precious metals in water electrolysis systems.

To better assess the catalytic advantage of fullerene-supported SACs, their key activity metrics were compared with conventional benchmark catalysts such as Pt/C and RuO_2_. As summarized in Table 1, Pt/C_60_ and V/C_60_ exhibit comparable or even superior intrinsic activity and stability relative to commercial benchmarks, confirming the potential of fullerene frameworks as efficient supports for SACs.

### 3.3. Structural Diversity and Coordination Complexity

However, research on fullerene-based SACs for electrocatalytic water splitting has only emerged in the past two years, with current studies predominantly focused on C_60_ related systems. The fullerene family actually encompasses remarkable diversity. The Computational Nanotechnology Lab at Michigan State University, led by Professors David Tomanek and Nick Frederick, has established an online database (https://nanotube.msu.edu/fullerene/fullerene-isomers.html, accessed on 7 November 2025) based on Professor M. Yoshida’s fullerene library, which catalogs three-dimensional structures of over 2487 known fullerene isomers.

**Figure 10:**
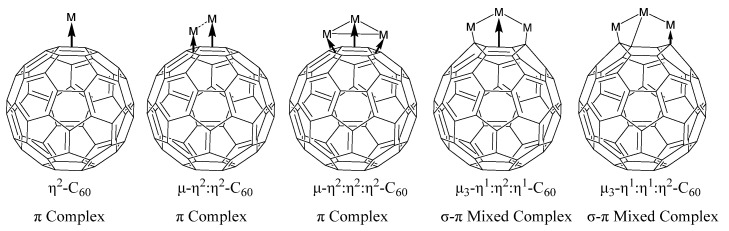
Possible architectures of metal-C_60_ composites.

Furthermore, reported studies have identified more than 60 metal species capable of interacting with fullerenes or nano-carbon spheres [26,27,28]. Consequently, the potential combinations of single metals with fullerene substrates exceed 150,000 possibilities. As shown in Figure 10, further considering different coordination possibilities, the combination number will approach 800,000 kinds. Recently, it has been reported that fullerene ligands coordinating with Pt^II^ centers can achieve the self-assembly synthesis of rigid binuclear cyclic compounds containing two fullerenes, which further increases the complexity of the structure of this type of material [29]. Habicher et al. [29] report the design and synthesis of a unique fullerene-containing supramolecular complex through platinum(II)-directed self-assembly. The study focuses on the construction of a dinuclear cyclophane that incorporates two C_60_ moieties, resulting in a rigid, symmetrical [2 + 2] macrocyclic structure. This work contributes to the broader goal of engineering fullerene-based molecular architectures with defined geometry and electronic functionality for applications in molecular electronics and host–guest chemistry. The authors first synthesized a hexakis-adduct of C_60_ functionalized with dipyridyl ligands through a series of cyclopropanation and substitution reactions. These pyridyl-appended fullerenes (ligand 2) were then reacted with a cis-configured platinum(II) complex, [cis-Pt(PEt_3_)_2_(OTf)_2_], under mild conditions. The resulting self-assembly yielded the cyclophane complex (1) in high yield, featuring two C_60_ units bridged via Pt-N coordination bonds in a D_2_h-symmetric framework. Spectroscopic characterization (^1^H, ^13^C, ^19^F, ^31^P NMR, and IR) and X-ray crystallography confirmed the successful formation and structural details of the cyclophane. Crystallographic analysis revealed a nearly planar macrocyclic architecture despite non-ideal internal angles at the platinum and carbon vertices. The two C_60_ cages act as structural end caps, and the central cavity—formed by two quaternary carbon atoms and two Pt(II) centers—exhibits tight confinement, suggesting limited guest-accessible volume but potential for π-π interactions. Bond angles and distances at the Pt centers and pyridine donors deviated slightly from ideal values, indicating strain accommodated by the macrocycle. Importantly, the self-assembly proceeds selectively without oligomer formation, demonstrating the precision of metal-directed organization in complex fullerene systems. This study showcases how transition metal coordination can be harnessed to organize multiple fullerene units into defined architectures, expanding the synthetic toolbox for fullerene-based supramolecular chemistry. The resulting dinuclear Pt-cyclophane structure offers a promising scaffold for exploring intermolecular charge transport, host–guest recognition, or further assembly into higher-order networks. It also highlights the potential of C_60_ ligands not only as functional π-systems but as structural elements in metallosupramolecular design. The above-mentioned dimensional disaster and combinatorial explosion make the structure space of fullerene-based SACs extremely vast, and it is almost impossible to screen out HER and OER catalysts with excellent performance through experimental trial and error.

### 3.4. Critical Comparison and Scalability Challenges of Synthesis Strategies

Despite the promising catalytic performance of fullerene-supported SACs, their large-scale application remains restricted by synthetic and structural limitations. The precise anchoring of single atoms on fullerene surfaces typically relies on low-yield solution-based reactions or vapor-phase deposition, which are difficult to scale while preserving atomic dispersion. Moreover, the intrinsic hydrophobicity and poor processability of fullerenes hinder their uniform integration into electrode structures, leading to reduced reproducibility and limited device compatibility [27,30].

Existing synthesis approaches can generally be classified as wet-chemical, vapor-phase, or polymerization-based routes, each presenting distinct advantages and drawbacks. Wet-chemical functionalization methods, such as hydroxylation, carboxylation, or amination [23], enhance fullerene solubility and enable coordination with metal precursors, yet often suffer from limited atomic precision and inconsistent yields. Vapor-phase or atomic-layer deposition offers better control of metal dispersion but requires sophisticated equipment and high-vacuum environments, making large-scale implementation costly. Polymerized fullerenes and fullerene-based covalent networks improve mechanical stability and electrical conductivity, providing robust frameworks for atom immobilization, although these processes frequently involve harsh reaction conditions and low overall efficiency.

From a scalability perspective, several challenges persist. The production of high-purity fullerene derivatives remains expensive compared with other carbon supports such as graphene or carbon nanotubes. In addition, fullerene-based SACs may undergo gradual structural degradation or atom migration under prolonged electrochemical operation, compromising catalytic durability. Their limited solubility and interfacial compatibility with conductive substrates further constrain the fabrication of uniform electrodes [17]. Consequently, developing cost-effective purification, recycling, and deposition techniques is crucial for translating laboratory-scale findings to industrial production.

To overcome these issues, recent research has focused on hybridization and in situ assembly strategies. Functionalized fullerenes, polymerized C60 frameworks, and fullerene–polymer composites have demonstrated improved processability, higher metal loading, and enhanced electrochemical durability. Electrochemical self-assembly and solution-phase reduction methods offer scalable alternatives for anchoring single atoms directly onto modified fullerene surfaces. While each synthesis route involves trade-offs between precision, scalability, and cost, integrating green chemistry principles with automated synthesis and machine learning-guided optimization may ultimately enable reproducible and industrially viable production of fullerene-based SACs.

## 4. Machine Learning and Computational Screening for Fullerene SACs

The rapid advancement of computational chemistry and artificial intelligence has made machine learning-driven catalyst discovery a viable solution to address these challenges. By training neural networks and machine learning models, combined with high-throughput screening, performance optimization, mechanistic interpretation, and data-driven materials discovery, this approach can significantly accelerate the entire workflow of catalyst development—from design and synthesis to characterization and application [30,31,32]. A collaborative effort between Meta Artificial Intelligence (AI) and Carnegie Mellon University researchers has yielded two significant advancements: (1) the Open Catalyst 2022 (OC22) dataset containing 62,331 carefully curated DFT calculations spanning diverse oxide materials and adsorption configurations, and (2) the development of the GemNet-OC machine learning model. This model successfully reproduces literature-reported adsorption energies and establishes critical OER scaling relationships, while providing actionable guidance for experimental investigations [33].

The research group led by Professor Jinlan Wang at Southeast University addressed the longstanding challenge of optimizing photocatalytic performance in nitrogen-rich covalent triazine frameworks (CTFs)—a class of materials whose structural complexity has hindered conventional design approaches. By developing an innovative graph neural network-based machine learning model, the team systematically screened 14,920 potential structures, identifying 45 high-performance candidates. This computational guidance enabled the subsequent experimental synthesis of novel CTF materials exhibiting exceptional photocatalytic hydrogen production rates [34]. Professor Edward Sargent’s research team at the University of Toronto implemented a machine learning-assisted computational pipeline to systematically evaluate 2070 metal oxide candidates. Through experimental validation, they identified Ru_0_._6_Cr_0_._2_Ti_0_._2_O_2_ as exhibiting superior durability, stability, and exceptional overpotential performance—demonstrating both enhanced stability and activity compared to conventional RuO_2_ benchmarks [35]. The research group led by Professor Jun Jiang at the USTC developed an innovative machine learning framework utilizing spectroscopic descriptors to establish structure–property relationships for molecules adsorbed on metal single-atom catalysts. By leveraging the continuous tunability of these spectroscopic descriptors, they achieved precise design of catalytic structures with tailored adsorption states [36].

**Figure 11:**
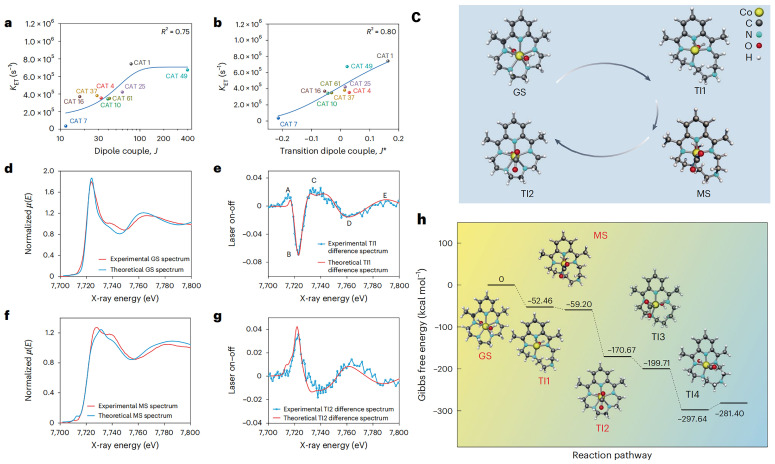
(**a**) Correlation of ET rate constant (KET) with intrinsic dipole coupling. (**b**) Correlation of KET with transition dipole coupling in nine PS–catalyst pairs. (**c**) Optimized structures of GS, TI, and MS of CAT 1. (**d**) Experimental and theoretical XANES spectra of CAT 1 at GS. (**e**) XANES difference spectrum of TI1 with theoretical fitting (delay 0.4  ±  0.3 µs, 7.1% fraction), where A, B, C, D, and E represent main positive absorption feature of TI1, minor positive absorption feature, main negative absorption feature of TI1, secondary negative feature and high-energy structural change feature, respectively. (**f**) Experimental and theoretical XANES spectra of CAT 1 at MS. (**g**) XANES difference spectrum of TI2 with theoretical fitting (delay 0.4 ± 0.3 µs, 4.5% fraction). (**h**) DFT-calculated free-energy profile of CAT 1 along the CO_2_RR pathway, with GS, TI1, TI2, and MS resolved by operando TR-XAS. Reproduced from Ref. [37] with permission from Springer Nature.

Building upon this work, Professor Jiang collaborated with Professor Yujie Xiong’s team at USTC to propose a machine learning-assisted high-throughput screening strategy. Jiang and Xiong’s teams present a machine learning-assisted high-throughput screening strategy to identify efficient molecular photocatalysts for CO_2_ reduction, integrating descriptor-based modeling with large-scale virtual screening. Unlike conventional trial-and-error or purely DFT-driven approaches, this framework combines quantum chemical calculations with data-driven models to accelerate catalyst discovery across vast chemical spaces. Specifically, thousands of candidate chromophore–catalyst dyads were constructed and their key physicochemical descriptors—such as frontier orbital energies, charge transfer integrals, and excited-state redox potentials—were computed as training inputs. Machine learning models were then trained to establish quantitative correlations between these descriptors and catalytic performance metrics, enabling rapid pre-screening of promising systems prior to explicit DFT validation. As shown in Figure 11, this approach successfully pinpointed optimal combinations that maximize light harvesting, electron transfer efficiency, and CO_2_ activation propensity. Importantly, the strategy revealed underlying design principles, highlighting, for example, the critical role of matching excited-state potentials with CO_2_ reduction energetics and balancing charge delocalization with molecular stability. Benchmarking against known photocatalysts confirmed the predictive power of the method, while newly identified dyads demonstrated significantly enhanced CO_2_-to-CO conversion under simulated solar irradiation. This work establishes machine learning-guided screening as a transformative methodology for molecular photocatalyst discovery, offering not only computational efficiency but also physically interpretable insights that can direct synthetic efforts toward high-performance CO_2_ reduction systems [37].

The research group led by Prof. Shaorui Sun at Beijing University of Technology combined DFT calculations with machine learning to investigate the adsorption free energies of hundreds of potential catalysts. They successfully identified 30 bimetallic site catalysts with superior ORR activity compared to Pt, 10 catalysts exhibiting better OER performance than RuO_2_, and 4 bifunctional catalysts demonstrating excellent activity for both ORR and OER [38]. Prof. Wanpeng Hu’s group at Jiaxing University systematically explored the performance of various single-metal-atom catalysts (TM@C_60_) in electrocatalytic two-electron oxygen reduction reaction (2e^−^ ORR) for hydrogen peroxide (H_2_O_2_) production using DFT and machine learning. Their machine learning approach revealed that the balance between O_2_ bond strength and adsorption energy is crucial for enhancing catalytic performance [39].

The aforementioned studies provide a crucial reference for catalyst development through the integration of theoretical calculations with machine learning. These studies demonstrate that this strategy can accurately predict the structural characteristics and adsorption properties of catalysts, facilitate the optimization of catalytic reaction conditions, and thereby enhance the performance and efficiency of catalysts.

DFT, through its electron density calculations, has proven highly effective for predicting surface–adsorbate interactions and reaction activity, making it indispensable in electrocatalysis research. However, current studies predominantly focus on adsorption simulations, while critically important transition state calculations remain both scarce and prone to significant errors. This represents a major knowledge gap, as transition states determine the reaction energy barriers core parameters governing reaction kinetics where calculation accuracy directly impacts the reliability of activation energy and reaction rate predictions [40].

**Figure 12:**
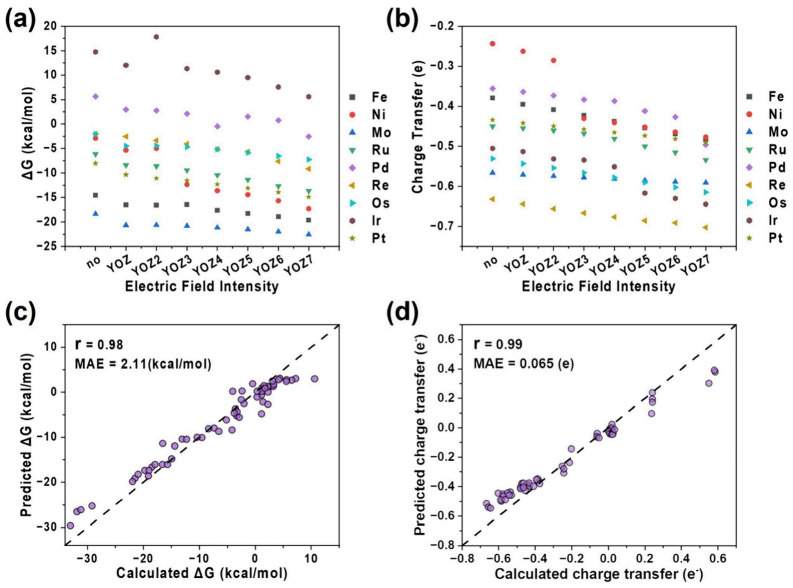
(**a**) Gibbs free energy of CO_2_ adsorption on M@g-C_3_N_4_ under varying electric fields. (**b**) Charge transfer of CO_2_ under different electric fields. (**c**) Predicted Gibbs free energy from IR and Raman spectra using attention neural networks. (**d**) Predicted charge transfer of CO_2_ from IR and Raman spectra using attention neural networks. Reproduced from Ref. [41] with permission from American Chemical Society.

To investigate the influence of potential on catalytic reactions, we conducted a systematic theoretical study to unravel the microscopic mechanisms by which external electric fields regulate CO_2_ electrocatalysis, proposing machine learning spectroscopy as a powerful bridge between spectral signals and catalytic properties [41. Using metal-doped graphitic C_3_N_4_ (M@g-C_3_N_4_) as a model system, they investigated CO_2_ adsorption across 27 single-atom catalysts under multiple directions and intensities of applied fields, thereby generating a comprehensive database encompassing nearly one thousand configurations. Traditional DFT analyses revealed diverse adsorption modes, from weak physical physisorption to strong carbon–oxygen chemisorption, and clarified how electric fields enhance adsorption energies, promote charge transfer, and distort CO_2_ molecular geometry to facilitate activation. However, the key innovation lies in coupling these electronic-structure results with machine learning models trained on infrared and Raman spectral descriptors. By employing convolutional neural networks with attention mechanisms, the authors successfully established a quantitative mapping between vibrational features and catalytic properties such as adsorption free energy and electron redistribution. Importantly, the models demonstrated not only high predictive accuracy but also interpretability: attention weights naturally converged on the asymmetric stretching vibrations of CO_2_, a spectroscopic fingerprint intrinsically linked to catalytic activation. Incorporating additional prior knowledge, such as HOMO-LUMO gaps and dipole moments, further reduced systematic errors, yielding robust predictions of adsorption energetics with chemical accuracy. As shown in Figure 12, this framework enabled not only forward prediction of catalytic behavior but also inverse determination of the electric field strength from spectroscopic data, providing a new paradigm for digitally monitoring and controlling electrocatalytic reactions. Beyond offering quantitative insights into the role of electric fields in CO_2_ reduction, this study highlights the broader potential of machine learning spectroscopy as a generalizable platform to uncover hidden structure–property relationships, accelerate catalyst screening, and enable real-time, in situ regulation of electrochemical processes under complex external stimulation [41].

Overall, the integration of machine learning with electronic-structure theory not only accelerates catalyst screening but also provides physically interpretable insights for guiding synthesis and operando characterization. Future efforts should focus on developing unified databases for fullerene-based SACs, integrating structural, spectral, and catalytic descriptors to enable transferable machine learning models across reaction systems. Furthermore, combining ML-driven prediction with automated experimental platforms will be crucial for realizing closed-loop catalyst discovery toward practical water electrolysis applications.

## 5. Conclusions and Perspective

Fullerene-based SACs have emerged as a highly promising class of materials for electrocatalytic overall water splitting. However, critical insights into their catalytic mechanisms—particularly those involving transition states—remain limited. Recent advances in theoretical modeling and machine learning now offer unprecedented opportunities to systematically unravel the complex structure–performance relationships governing these catalysts. Looking forward, the development of a robust high-throughput computational framework capable of screening tens of thousands of fullerene isomers doped with a broad spectrum of metal atoms will be indispensable. Such a framework will enable comprehensive potential energy surface optimization, facilitating the identification of all relevant local minima and transition states. By incorporating reaction path calculations under variable electric field conditions, more accurate kinetic descriptions can be achieved, deepening our understanding of the electrocatalytic process at the atomic level.

Nevertheless, several experimental challenges remain to be addressed before fullerene-based SACs can be translated into practical water-splitting devices. First, the precise atomic configuration and coordination environment of metal centers on fullerene supports are still difficult to control experimentally, especially under electrochemical operating conditions. The lack of in situ or operando spectroscopic characterization techniques limits direct observation of dynamic active sites during catalytic turnover. Second, scalable synthesis of uniform fullerene–metal architectures with high loading and stability remains a formidable challenge, as most reported methods rely on low-yield, solution-phase reactions. Third, the long-term durability and electrochemical stability of fullerene-supported catalysts under realistic bias and pH conditions have not been systematically evaluated. Furthermore, the interaction between fullerene curvature, spin polarization, and charge transport pathways is yet to be fully understood experimentally.

**Figure 13:**
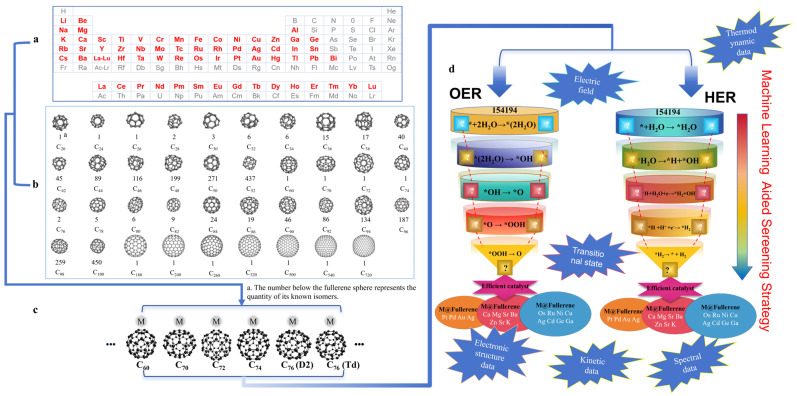
(**a**) Metals that may form fullerene-based SACs. (**b**) The reported numbers of fullerenes and their isomers (numbers shown beneath the fullerene structures). (**c**) Several representative fullerene-based SACs. “*” represent the adsorbed states d Machine learning aided screening strategy.

Future research should therefore emphasize the integration of advanced experimental and computational tools. Combining operando X-ray absorption spectroscopy, time-resolved photoelectron spectroscopy, and scanning transmission electron microscopy can provide direct structural and electronic insights at the atomic level. Parallelly, as shown in Figure 13, the establishment of a large-scale, standardized database linking structural and electronic descriptors of fullerene-based SACs to their catalytic performance will further accelerate data-driven discovery. Leveraging advanced machine learning algorithms to map atomic-scale features to key performance metrics—such as activation energies, adsorption energies, and overpotentials—will allow the creation of intelligent predictive models. These models have the potential to significantly shorten the design cycle and guide the rapid screening of high-performance SACs for water splitting. Furthermore, integrating these computational tools with emerging “machine chemist” platforms can enable a seamless full-cycle research workflow—from structure prediction to experimental synthesis—of novel fullerene-based electrocatalysts. Realizing this vision will require close collaboration between computational scientists and experimentalists to ensure that theoretical predictions translate into practical catalytic systems.

In summary, the synergistic integration of advanced theoretical calculations, machine learning, and experimental validation is poised to drive transformative progress in the rational design of fullerene-based SACs, paving the way for efficient and scalable hydrogen production technologies that contribute to a sustainable energy future.

## Figures and Tables

**Table 1:** Comparative HER/OER performance metrics for representative fullerene-based catalysts and benchmark catalysts (conditions: 1.0 M KOH unless otherwise noted).

Catalyst (Ref.)	Electrolyte/Conditions	HER η10 (mV)	Tafel Slope (mV·dec^−1^)	Mass Activity/TOF/ECSA-Normalized Data	Stability/Remarks
Pt single atoms on C_60_ (Pt/C_60_–2) [18]	1.0 M KOH, 90% iR-corrected	25	55	TOF: 2.17 s^−1^ (50 mV), 5.55 s^−1^ (100 mV), 11.2 s^−1^ (150 mV); higher mass activity than 20 wt% Pt/C	Stable for 100 h at 10 mA·cm^−2^ (Δη ≈ +31.5 mV); negligible decay after 3000 CV cycles
Ru nanoparticles on fullerenol (Ru–OC60–300) [23]	1.0 M KOH	4.6	24.7	High mass activity; ECSA/TOF reported in SI	Excellent durability and Faradaic efficiency; η depends on loading and iR correction
C_60_-supported V single atom (mechanistic) [19,20,21]	Gas-phase/IRMPD + DFT	-	-	Mechanistic study; >70 kJ·mol^−1^ barrier reduction, H-shuttle effect observed	Not directly comparable to bulk HER/OER performance
Commercial 20 wt% Pt/C (benchmark)	1.0 M KOH	≈39	≈99	Standard benchmark; mass activity and TOF vary with loading	Common reference catalyst; see Ref. [18] for comparison
RuO_2_ (benchmark for OER)	1.0 M KOH	n.a.	n.a.	OER η10 typically 200–350 mV (literature range)	Widely used benchmark; stability depends on morphology

## Data Availability

No new data were created or analyzed in this study. Data sharing is not applicable to this article.

## References

[1] Gunathilake C., Soliman I., Panthi D., Tandler P., Fatani O., Ghulamullah N.A., Marasinghe D., Farhath M., Madhujith T., Conrad K. (2024). A comprehensive review on hydrogen production, storage, and applications. Chem. Soc. Rev..

[2] Zhang D., Li M., Yong X., Song H., Waterhouse G.I.N., Yi Y., Xue B., Zhang D., Liu B., Lu S. (2023). Construction of Zn-doped RuO_2_ nanowires for efficient and stable water oxidation in acidic media. Nat. Commun..

[3] Tang J., Guan D., Xu H., Zhao L., Arshad U., Fang Z., Zhu T., Kim M., Pao C.-W., Hu Z. (2025). Undoped ruthenium oxide as a stable catalyst for the acidic oxygen evolution reaction. Nat. Commun..

[4] Liang C., Rao R.R., Svane K.L., Hadden J.H.L., Moss B., Scott S.B., Sachs M., Murawski J., Frandsen A.M., Riley D.J. (2024). Unravelling the effects of active site density and energetics on the water oxidation activity of iridium oxides. Nat. Catal..

[5] Zhang J., Fu X., Kwon S., Chen K., Liu X., Yang J., Sun H., Wang Y., Uchiyama T., Uchimoto Y. (2025). Water splitting by C_6__0_-Supported Vanadium Single Atoms. Science.

[6] Zhao J., Guo Y., Zhang Z., Zhang X., Ji Q., Zhang H., Song Z., Liu D., Zeng J., Chuang C. (2025). Out-of-plane coordination of iridium single atoms with organic molecules and cobalt–iron hydroxides to boost oxygen evolution reaction. Nat. Nanotechnol..

[7] Feidenhans’l A.A., Regmi Y.N., Wei C., Xia D., Kibsgaard J., King L.A. (2024). Precious Metal Free Hydrogen Evolution Catalyst Design and Application. Chem. Rev..

[8] Park Y., Jang H.Y., Lee T.K., Kim T., Kim D., Kim D., Baik H., Choi J., Kwon T., Yoo S.J. (2025). Atomic-level Ru-Ir mixing in rutile-type (RuIr)O_2_ for efficient and durable oxygen evolution catalysis. Nat. Commun..

[9] Zhang S., He X., Ding Y., Shi Z., Wu B. (2024). Supply and demand of platinum group metals and strategies for sustainable management. Renew. Sust. Energy Rev..

[10] Gao Z., Li A., Liu X., Peng M., Yu S., Wang M., Ge Y., Li C., Wang T., Wang Z. (2025). Shielding Pt/γ-Mo_2_N by inert nano-overlays enables stable H_2_ production. Nature.

[11] Hou L., Cui X., Guan B., Wang S., Li R., Liu Y., Zhu D., Zheng J. (2022). Synthesis of a monolayer fullerene network. Nature.

[12] Peng B.J. (2022). Monolayer fullerene networks as photocatalysts for overall water splitting. Am. Chem. Soc..

[13] Wu J., Peng B.J. (2025). Smallest [5,6] fullerene as building blocks for 2D networks with superior stability and enhanced photocatalytic performance. Am. Chem. Soc..

[14] Kroto H.W., Heath J.R., O’Brien S.C., Curl R.F., Smalley R.E. (1985). C_6__0_*:* Buckminsterfullerene. Nature.

[15] Wang T., Zhang L., Wu J., Chen M., Yang S., Lu Y., Du P. (2023). Few-Layer Fullerene Network for Photocatalytic Pure Water Splitting into H_2_ and H_2_O_2_. Angew. Chem. Int. Ed..

[16] Kment Š., Bakandritsos A., Tantis I., Kmentová H., Zuo Y., Henrotte O., Naldoni A., Otyepka M., Varma R.S., Zbořil R. (2024). Single Atom Catalysts Based on Earth-Abundant Metals for Energy-Related Applications. Chem. Rev..

[17] Cao D., Zhang Z.-R., Cui Y.-H., Zhang R., Zhang L., Zeng J., Cheng D. (2023). One-Step Approach for Constructing High-Density Single-Atom Catalysts toward Overall Water Splitting at Industrial Current Densities. Angew. Chem. Int. Ed..

[18] Zhang R., Li Y., Zhou X., Yu A., Huang Q., Xu T., Zhu L., Peng P., Song S., Echegoyen L. (2023). Single-atomic platinum on fullerene C_6__0_ surfaces for accelerated alkaline hydrogen evolution. Nat. Commun..

[19] Hou G.-L., Yang T., Li M., Vanbuel J., Lushchikova O.V., Ferrari P., Bakker J.M., Janssens E. (2021). Water Splitting by C_6__0_-Supported Vanadium Single Atoms. Angew. Chem. Int. Ed..

[20] Xu J., Bakker J.M., Lushchikova O.V., Lievens P., Janssens E., Hou G.-L. (2023). Pathways of cluster growth: Infra-red multi-photon dissociation spectroscopy of a series of Re–Si clusters, [ReSi_*n*_]^+^, n = 3–9. J. Am. Chem. Soc..

[21] Li M., Yang T., Bakker J.M., Janssens E., Hou G.-L. (2022). Unveiling the role of C_6__0_-supported vanadium single atoms for catalytic overall water splitting. Cell Rep. Phys Sci..

[22] Lee K., Song H., Park J.T. (2003). [60] Fullerene-Metal Cluster Complexes: Novel Bonding Modes and Electronic Communication. Acc. Chem. Res..

[23] Li Y., Xu T., Huang Q., Zhu L., Yan Y., Peng P., Li F.-F. (2023). C_60_ Fullerenol to Stabilize and Activate Ru Nanoparticles for Highly Efficient Hydrogen Evolution Reaction in Alkaline Media. ACS Catal..

[24] Wang X., Yang Q., Singh S., Borrmann H., Hasse V., Yi C., Li Y., Schmidt M., Li X., Fecher G.H. (2025). Topological semimetals with intrinsic chirality as spin-controlling electrocatalysts for the oxygen evolution reaction. Nat. Energy.

[25] Lu Z., Ronson T.K., Heard A.W., Feldmann S., Vanthuyne N., Martinez A., Nitschke J.R. (2023). Enantioselective fullerene functionalization through stereochemical information transfer from a self-assembled cage. Nat. Chem..

[26] Balch A.L., Winkler K. (2021). Electrochemistry of fullerene/transition-metal complexes: Three decades of progress. Coord. Chem. Rev..

[27] Yamada M., Akasaka T., Nagase S. (2010). Endohedral metal atoms in pristine and functionalized fullerene cages. Acc. Chem. Res..

[28] Hu Z., Yang S. (2024). Endohedral metallofullerene molecular nanomagnets. Chem. Soc. Rev..

[29] Habicher T., Nierengarten J.-F., Gramlich V., Diederich F. (1998). PtII-Directed Self-Assembly of a Dinuclear Cyclophane Containing Two Fullerenes. Angew. Chem. Int. Ed..

[30] Ding R., Chen J., Chen Y., Liu J., Bando Y., Wang X. (2024). Unlocking the potential: Machine learning applications in electrocatalyst design for electrochemical hydrogen energy transformation. Chem. Soc. Rev..

[31] Butler K.T., Davies D.W., Cartwright H., Isayev O., Walsh A. (2018). Machine learning for molecular and materials science. Nature.

[32] Merchant A., Batzner S., Schoenholz S.S., Aykol M., Cheon G., Cubuk E.D. (2023). Scaling deep learning for materials discovery. Nature.

[33] Tran R., Lan J., Shuaibi M., Wood B.M., Goyal S., Das A., Heras-Domingo J., Kolluru A., Rizvi A., Shoghi N. (2023). The Open Catalyst 2022 (OC22) Dataset and Challenges for Oxide Electrocatalysts. ACS Catal..

[34] Wu M., Song Z., Cui Y., Fu Z., Hong K., Li Q., Lyu Z., Liu W., Wang J. (2025). Machine Learning-Assisted Design of Nitrogen-Rich Covalent Triazine Frameworks Photocatalysts. Adv. Funct. Mater..

[35] Abed J., Heras-Domingo J., Sanspeur R.Y., Luo M., Alnoush W., Meira D.M., Wang H., Wang J., Zhou J., Zhou D. (2024). Pourbaix Machine Learning Framework Identifies Acidic Water Oxidation Catalysts Exhibiting Suppressed Ruthenium Dissolution. J. Am. Chem. Soc..

[36] Yang T., Zhou D., Ye S., Li X., Li H., Feng Y., Jiang Z., Yang L., Ye K., Shen Y. (2023). Catalytic Structure Design by AI Generating with Spectroscopic Descriptors. Am. Chem. Soc..

[37] Hu Y., Yu C., Wang S., Wang Q., Reinhard M., Zhang G., Zhan F., Wang H., Skoien D., Kroll T. (2025). Identifying a highly efficient molecular photocatalytic CO_2_ reduction system via descriptor-based high-throughput screening. Nat. Catal..

[38] Fang Z., Li S., Zhang Y., Wang Y., Meng K., Huang C., Sun S.J. (2024). The DFT and Machine Learning Method Accelerated the Discovery of DMSCs with High ORR and OER Catalytic Activities. Phys. Chem. Lett..

[39] Cao Y., Liu T., Chen J., Cai S., Liu J., Huang H., Zhong W., Meng Y., Zhang R., Xia Q. (2024). Screening of highly efficient electrocatalysts for hydrogen peroxide synthesis using single transition metal atoms embedded in carbon vacancy fullerene C_60_. Chem. Eng. Sci..

[40] Araujo R.B., Rodrigues G.L.S., dos Santos E.C., Pettersson L.G.M. (2022). Adsorption energies on transition metal surfaces: Towards an accurate and balanced description. Nat. Commun..

[41] Cui C.X., Shen Y., He J.R., Fu Y., Hong X., Wang S., Jiang J., Luo Y.J. (2024). Quantitative Insight into the Electric Field Effecton CO_2_ Electrocatalysis via Machine Learning Spectroscopy. Am. Chem. Soc..

